# A Brief Overview on BDNF-Trk Pathway in the Nervous System: A Potential Biomarker or Possible Target in Treatment of Multiple Sclerosis?

**DOI:** 10.3389/fneur.2022.917527

**Published:** 2022-07-12

**Authors:** Giuseppe Schirò, Salvatore Iacono, Paolo Ragonese, Paolo Aridon, Giuseppe Salemi, Carmela Rita Balistreri

**Affiliations:** ^1^Unit of Neurology, Department of Biomedicine, Neurosciences and Advanced Diagnostics, University of Palermo, Palermo, Italy; ^2^Cellular and Molecular Laboratory, Department of Biomedicine, Neuroscience and Advanced Diagnostics (Bi.N.D.), University of Palermo, Palermo, Italy

**Keywords:** neurodegenerative disorders, multiple sclerosis, BDNF-Trk pathway, biomarkers, targets

## Abstract

The growing incidence of neurodegenerative disorders in our populations is leading the research to identify potential biomarkers and targets for facilitating their early management and treatments. Biomarkers represent the crucial indicators of both physiological and pathological processes. Specific changes in molecular and cellular mechanisms of physiological processes result in biochemical alterations at systemic level, which can give us comprehensive information regarding the nature of any disease. In addition, any disease biomarker should be specific and reliable, able to consent of distinguishing the physiological condition of a tissue, organ, or system from disease, and be diverse among the various diseases, or subgroups or phenotypes of them. Accordingly, biomarkers can predict chances for diseases, facilitate their early diagnosis, and set guidelines for the development of new therapies for treating diseases and disease-making process. Here, we focus our attention on brain neurotrophic factor (BDNF)–tropomyosin receptor kinase (Trk) pathway, describing its multiple roles in the maintenance of central nervous system (CNS) health, as well as its implication in the pathogenesis of multiple sclerosis (MS). In addition, we also evidence the features of such pathway, which make of it a potential MS biomarker and therapeutic target.

## Introduction

The increase in incidence of neurodegenerative disorders is leading to search potential biomarkers and targets for facilitating their management and treatments. The particular focus on related pathophysiological mechanisms and pathways can consent to achieve this important aim. Among these, the alterations in expression of neurotrophins are emerging (1). Here, we report a brief overview on brain neurotrophic factor (BDNF)–tropomyosin receptor kinase (Trk) pathway, describing its numerous roles in the maintenance of central nervous system (CNS) health, as well as its implication in the pathogenesis of multiple sclerosis (MS). Our aim was to provide an updated and comprehensive evidence regarding the role of BDNF-Trk pathway in MS pathology with an emphasis on the probability that its expression and levels could represent a potential biomarker and target.

### BDNF: Molecular Characteristics and Functions

Brain neurotrophic factor (BDNF) is a neurotrophin, a member of a large family of neurotrophins, including the nerve growth factor (NGF), neurotrophin-3 (NT3), and neurotrophin-4 (NT4) (2). BDNF plays an important role in maintaining the structural integrity and function of neurons, influencing their growth, survival, and differentiation. The expression of BDNF has been documented both in the CNS—where it is the most abundant neurotrophic factor (3)—and the peripheral nervous system (PNS) (4). Furthermore, BDNF is produced by the neurons and oligodendrocytes, but also platelets (4, 5), cells of the immune system (i.e., T and B lymphocytes, monocytes/macrophages) (6), and active muscles can release BDNF, therefore representing the main reserve of BDNF at the peripheral level (7).

The *BDNF* gene is located on chromosome 11 (11p14.1), between the loci of the *FSHB* and *HVBS1* genes, in a region of about 4 Mb (7). In both humans and rodents, it contains nine exons, each of which has its own promoter; because of this, many kinds of transcripts are known, even if the final product of the translation is then identical for all (8). The existence of different promoters is, however, very important in terms of temporal and spatial regulation since different promoters can be used in different brain regions and cell types. Some variants of transcripts, deriving from four coding exons, are expressed at the cardiac and pulmonary level (9). Overall, the *BDNF* gene can produce about 34 different transcripts in response to a wide variety of stimuli (10), which are polyadenylated at the level of two alternative sites, leading to the formation of two populations of mRNA: one with a short untranslated region (UTR) at the 3 “end, and the other with a long UTR at the 3” end (9). The two types of transcripts are also characterized by a different localization: sequences with a short UTR at the 3 “end are restricted to the cell soma, and sequences with a long UTR at the 3” end can also be found in dendrites for local translation (11).

Likewise, the synthesis of BDNF also is a complex process. The polypeptide is synthesized starting from a precursor, the pre-pro-BDNF at the level of the endoplasmic reticulum (12). Subsequently, the removal of the leader peptide takes place, with the formation of pro-BDNF, a protein weighing 32 kDa. The pro-BDNF, in turn, undergoes a proteolytic cut that generates, within the cell, the mature BDNF (13). However, pro-BDNF can also be cut later, after secretion in the extracellular environment, by the plasmin serine protease (14) and matrix metalloproteases (15) and it is also endowed with bioactive actions on the cells. It is interesting to note how the two forms, the immature form, called pro-BDNF, and the mature form, the BDNF, have not only different binding properties (16), but also multiple diversity in biological functions. In fact, BDNF promotes neuronal survival, cell differentiation, synaptic plasticity, and long-term potentiation (LTP). The pro-BDNF could induce apoptosis, reduces the density of dendritic spines, determines the retraction of the cones of growth, and facilitates long-term depression (LTD) at the hippocampal level (17).

### Focus on BDNF-Trk Pathway

**Figure 1:**
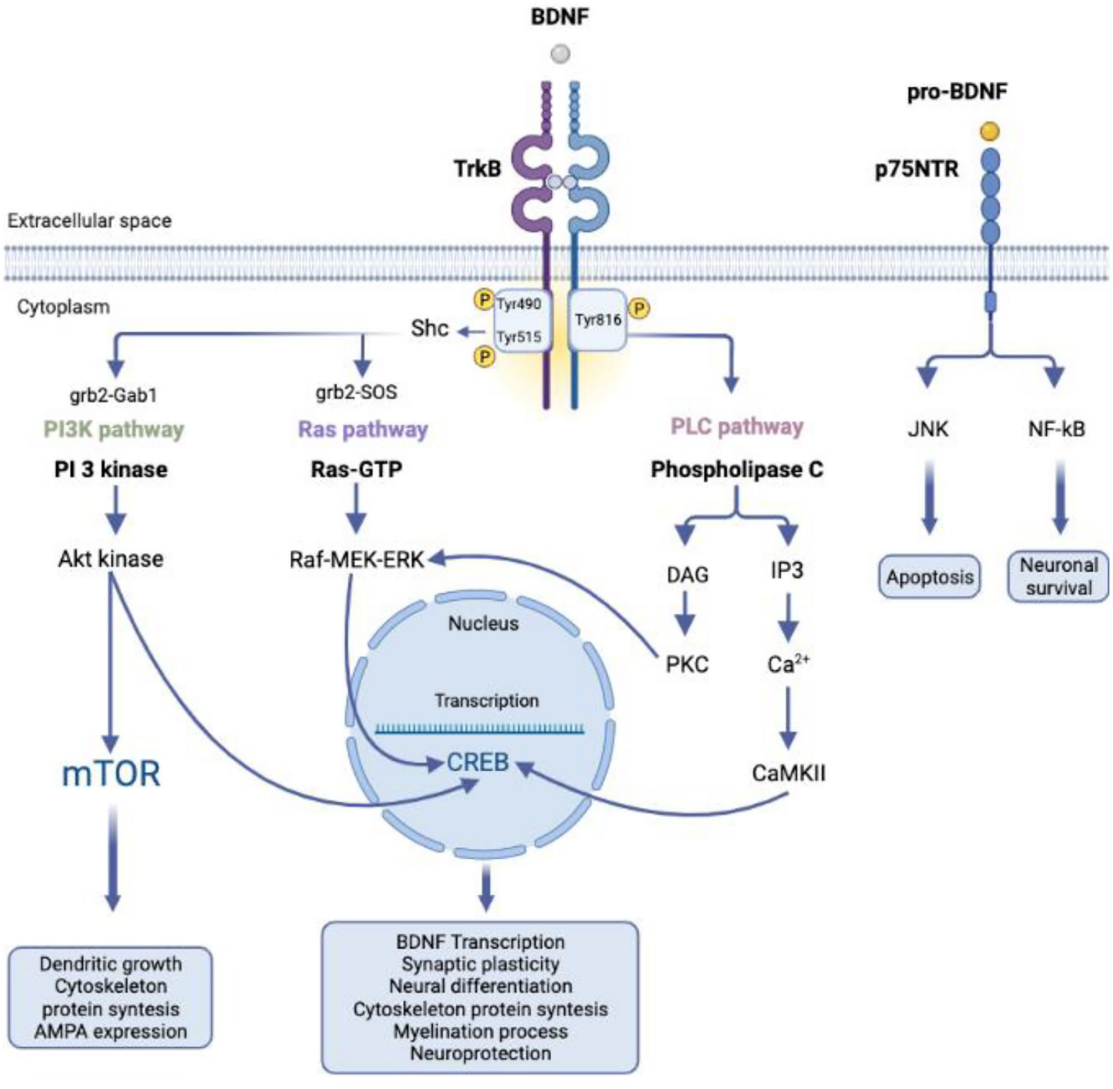
TrkB and p75NTR signaling. The binding between BDNF and TrkB induces the activation of three transduction pathways at the intracellular level mediating their effects on neuronal growth and synaptic plasticity. The interaction between pro-BDNF and p75NTR receptor promotes cell apoptosis and neuronal survival via JNK NF-kB, respectively. BDNF, brain-derived neurotrophic factor; TrkB, tropomyosin receptor kinase; p75NTR, p75 neurotrophin receptor; Shc, Grb2, growth factor receptor-bound protein 2; SOS, Son of Sevenless; Gab1, GRB2-associated-binding protein 1; AKT, protein kinase B; PI3K, phosphatidylinositol 3-kinase; PLC, phospholipase C; PKC, protein kinase C; DAG, diacylglycerol; IP3, inositol-3-phosphate; CaMKII, type II calcium/calmodulin-dependent protein kinase; JNK, c-Jun N-terminal kinases; NF-kB, nuclear factor kappa B; CREB, cAMP response element-binding protein; mTOR, mammalian target of rapamycin; AMPA, α-Ammino-3-idrossi-5-Metil-4-isossazol-Propionic Acid.

BDNF mediates its effects by interacting with two types of receptors: The first belongs to the family of receptors with tyrosine kinase activity namely tropomyosin receptor kinase (i.e., Trk), while the second is a neurotrophin receptor with low binding affinity, known as p75 neurotrophin receptor (i.e., p75NTR) (Figure 1).

The class of Trk receptors includes three isoforms, TrkA, TrkB, TrkC, resulting from different mRNA splicing events (18, 19). Although they are highly conserved and have numerous homologous sequences, Trk receptors differ in the extracellular domain, responsible for the interaction with ligands. This makes each isoform specific for a different neurotrophin. In detail, TrkA is activated by NGF (20), TrkB is activated by BDNF (21) and NT4 (22), and TrkC is activated by NT3. However, in some stages of brain development, NT-3 can also activate TrkA and TrkB (23).

The interaction of BDNF with TrkB can induce the activation of three different signal transduction pathways at the intracellular level: the phospholipase-Cγ (PLCγ) pathway, the phosphatidylinositol 3-kinase (PI3K) pathway and the kinase pathway regulated by extracellular signals (ERK), and members of the mitogen-activated protein kinase (MAPK) family (24) (Figure 1). Once BDNF is bound, TrkB undergoes dimerization and phosphorylation on the intracytoplasmic kinase side (25). Phosphorylation on Y816 activates PLCγ (26) with increased intracellular calcium and activation of type II calcium/calmodulin-dependent protein kinase (CaMKII). The result is the activation of the CREB transcription factor. On the contrary, the phosphorylation on residue Y490 facilitates the recruitment of Shc (27) which, once phosphorylated, activates the Grb2/SOS complex. SOS, in proximity of the plasma membrane, induces the detachment of the GDP from the Ras protein, which can therefore bind to the GTP, activating itself. Activated Ras, in turn, initiates the cascade that recruits Raf, MEK, and ERK in succession (28). Instead, the activation of PI3K and, further downstream, of the Akt kinase occurs starting from the bond of Grb2 with Gab1. Akt and ERK can also activate CREB or mTOR, which, respectively, promote gene expression and protein translation (29).

The TrkB receptor exists in two isoforms, the gp145TrkB and the truncated gp95TrkB, characterized by identical transmembrane and extracellular structure and the absence of the tyrosine kinase domain. The transcripts for gp145TrkB have been identified at the level of the motor cortex and pyramidal neurons of the hippocampus, while the transcripts for gp95TrkB are located at the level of the ependymal lining of the cerebral ventricles and in the choroid plexuses (21). In MS, the expression of the whole form of TrkB appears to contribute to the maintenance of autoimmunity of peripherally recruited cells, mediating the resistance of T lymphocytes to apoptosis induced by the so-called “activation-induced cell death mechanism” (AICD) (30).

Both BDNF and TrkB are widely expressed in the CNS, especially in the cerebral cortex, hippocampus, and cerebellum (31). The presence of BDNF has also been demonstrated in the striatum, synthesized at the cortical level, and released by the descending cortico-striatal pathways (32). In cortical neurons, BDNF production appears to be regulated by NT-4/5, as well as by BDNF itself, in a glutamate receptor-dependent manner. Through TrkB, BDNF and NT-4/5 activate dose-dependent mRNA production for BDNF, suggesting that neurotrophic factors are capable of mutual modulation. Stimulation of TrkB leads to the activation of MAPK and PI3K, which negatively and positively modulate, respectively, the expression of AMPA receptors. In turn, AMPA receptors increase the expression of the BDNF gene through the activation of Lyn, a member of the Src family, capable of activating MAPK with a consequential increase in BDNF (33).

The p75^NTR^ receptor, on the contrary, belongs to the superfamily of TNF-α receptors, is encoded by a gene composed of 10 exons and 11 introns (34), and consists of an extracellular domain, a transmembrane domain, and an intracellular domain. The extracellular domain has four cysteine repeats, and the cysteines in positions three and four are essential for the interaction with the ligands (35). The intracellular domain, known as the death domain, is capable both *in vivo* and *in vitro* of causing the death of nerve cells. If hyper-expressed and in multimeric forms, p75^NTR^ can cause cell death regardless of binding to a ligand, presumably because of spontaneous activation of the intracellular signal from the death domain (36) and the activation of the JNK kinase (Jun N-terminal kinase). Despite the expression of p75^NTR^ often correlates with neuronal death, multiple evidence indicates its involvement in cell survival mechanisms. In fact, in some cases, the absence of the p75^NTR^ receptor leads to an increase in the death of peripheral sensory neurons (37). It has been hypothesized that this may be due to either the death or impaired migration of Schwann cells, which are necessary for the survival of sensory neurons. Among other things, p75^NTR^ is expressed at high levels by Schwann cells to ensure their correct migration along the peripheral nerves (38, 39).

Furthermore, p75^NTR^ can activate, alternatively to JNK, NF-κB, thus promoting cell survival (40).

Ultimately, p75^NTR^ can mediate both death and cell survival. Its final effect therefore depends on the context and on the different and numerous pathways activated in each cell (41).

## Effects of BDNF in the Nervous System

### BDNF Role in the Myelination Process

Several evidence from animal studies consistently indicated the contribution of BDNF pathways to the myelination process. In mice, the impaired expression of p75^NTR^ results in reduced myelination at the SNP level, in the absence of alterations on myelination of the CNS. It has been suggested that the p75^NTR^ receptor is the one responsible for BDNF-induced myelin synthesis at the SNP level (42). On the contrary, BDNF seems to exert its myelinating effect on the CNS via the receptor TrkB (43). In a cuprizone-induced mouse model of demyelination, a positive correlation was noted between BDNF levels and myelin expression by oligodendrocytes (44). BDNF appears in fact to induce myelination by oligodendrocytes through the ERK1/2 phosphorylation (90). Other evidence arrives from BDNF-deficient mice expressing low levels of myelin proteins during the recovery phase of demyelination (45). More precisely, myelin basic protein (MBP) mRNA levels are significantly reduced in the hippocampus and cerebral cortex when BDNF gene expression is silenced (46). The activity of BDNF on oligodendrocytes is not limited anyway only to the induction of their functional activity. In fact, BDNF seems to be able to control their proliferation and differentiation. Accordingly, these processes can be hindered by a deficit in the BDNF production (47). Consistent with this evidence, relevant are the numerous data obtained from the group of Dr. Dreyfus in mice, animal, and culture works. Precisely, they have demonstrated that the BDNF levels impact oligodendrocyte lineage cells (47). In oligodendrocyte progenitor cells (OPCs), in culture, they have demonstrated that the reduced levels of BDNF influence the proliferation of OPCs (48). Adequate BDNF amounts, through the TrkB receptor, increase, indeed, the DNA synthesis in OPCs and induce differentiation of post-mitotic oligodendrocytes (OLGs) of the basal forebrain (BF). In addition, by using BDNF knockout animals, they have investigated the BDNF's effects on OLG *in vivo* (48). OLCs of BF resulted to express the TrkB receptor, suggesting their responsiveness to BDNF. Furthermore, immunohistochemistry analysis with NG2 and CC1 antibodies has been performed for evaluating the numbers of NG2+ OPCs and CC1+ post-mitotic BF OLGs in the embryo (E17) of both BDNF –/– and BDNF ± mice. A reduced number of NG2+ cells characterized the embryos without BDNF. The same result has been obtained in the BDNF ± mice at E17 and at postnatal day 1 (P1), P14, and adult, indicating the BDNF crucial role in OPC development (47). Accordingly, BDNF ± mice did not have an altered number of CC1+ OLGs, even if they showed decreased levels of myelin basic protein (MBP), myelin-associated glycoprotein (MAG), and proteolipid protein (PLP). Such data have consequently confirmed that BDNF also has a fundamental role in OLG differentiation, as well as in the impairment, in *vivo* and at decreased levels, of progenitor cells and myelin proteins (47).

### Modulation of Central Motor Structures by BDNF

The mRNA for BDNF is widely expressed in numerous neurons of the cerebellum, basal nuclei, brainstem, motor cortex, and spinal cord (32). These are areas with strategic functions on motor control and behavior influencing, and regulating the initiation, learning, execution, and coordination of motor activity. For example, at the cerebellar level, BDNF acts as a survival, differentiation, and morphogenetic development factor of Purkinje cells and cerebellar granules (49) and can induce rapid depolarization in cells of Purkinje through the activation of sodium channels (50). BDNF deprivation in the striatum leads to the loss of numerous dopaminergic neurons. On the contrary, following a spinal injury, treatment with BDNF prevents the death of spinal motor neurons *in vivo* and can promote the connections of the corticospinal tract with the damaged cord (51). It seems that BDNF can also mediate the activation of anti-apoptotic pathways at the spinal level by recruiting ERK. Activation of PI3K/Akt may also determine the same effect, thanks to the production of BAD, an inhibitor of NF-κB (nuclear factor kappa-light-chain-enhancer of activated B cells), and of the transcription factor FKHRL1(52). Furthermore, BDNF stimulates post-synaptic excitatory potentials (EPSPs) via NMDA receptors on spiny neurons of adult mouse striatum and induces LTP production in cortico-striatal synapses (53).

### BDNF Modulation of Synaptic Plasticity and Activity

BDNF stimulates the increase in the density of synaptic spines through a mechanism dependent on the Ras/ERK pathway (54) and the activation of the TRPC (transient receptor-potential cation channel subfamily C) type 3 ion channel (55). At the hippocampal level, BDNF induces the polymerization of actin filaments in dendritic spines through the modulation of PAK (p21-activated kinase) and ADF (actin-depolymerizing factor)/cofilin, determining the initiation and maintenance of LTP *in vivo* (long-term potentiation) (56). In the context of the LPT, BDNF does not appear to act directly at the post-synaptic level but appears to act positively on the pre-synaptic terminals, preparing the exocytosis events necessary to modify the responses of the post-synaptic neurons (56). Moreover, many of the functions of BDNF on synaptic plasticity depend on its ability to modulate the expression levels of numerous micro-RNAs. For example, long-term stimulation by BDNF regulates the extension and branching of axons in cortical neuronal cultures by increasing the levels of mir-9 (57). Another miRNA capable of responding to BDNF is miR-132, which is induced by BDNF via CREB activation and induces dendritic growth, inhibiting translation of the P250GAP protein (58). Inhibition of mir-132 causes a decrease in the BDNF-dependent expression of important synaptic proteins such as GluN2A, GluN2B, and GluA1 (59).

In animal model of MS, such as EAE, the administration of IL-17 exerts immune-protective effects through the reduction in mir-155 levels with a parallel increase in BDNF levels (60). In the PMBC of patients with symptomatic relapsing–remitting MS, miR-132-3p, miR-106b-5p, and miR-19b-3p are downregulated and their levels are related to BDNF levels (61).

### BDNF and Neuroprotection

The neuro-protective activity is ensured by several mechanisms. In hippocampal neurons, BDNF eliminates glutamatergic toxicity via ERK and PI3K signaling (62). In primary rat cortical cultures, the effects of BDNF against apoptosis and mitochondrial dysfunction induced by 3-nitropropionic acid (3-NP) are mediated through nitric oxide (NO), cyclic guanosine monophosphate (cGMP)-dependent protein kinase (PKG), and NF-κB (63). Neuroprotection and reduction in neuroinflammation in numerous animal and cellular studies are also often accompanied by an increase in BDNF expression. For example, fingolimod, a sphingosine-1-phosphate receptor modulator used to treat MS, causes increased BDNF expression in experimental models of Huntington's disease, MS, and AD (64–66).

### Effect on Cell Survival, Migration, and Differentiation

BDNF has several effects on cell development. For example, BDNF is a migration and differentiation factor of cortical GABAergic inter-neurons, regulating their development by stimulating growth and ensuring the stabilization of synapses. This function is performed in synergy with the endocannabinoid system (eCS) that enhances the morphogenetic effects of BDNF through CB1-TrkB trans-activation (67). In general, BDNF seems to play a role in promoting the development of different cell subpopulations: It, in fact, stimulates neuronal differentiation and the growth of dendrites in the neurons of the sub-granular zone in the hippocampus. In this region, it seems that a decrease in BDNF levels does not affect the number of neurons but may compromise synaptic plasticity (68). BDNF is essential also for the development, growth, and differentiation of sensory neurons starting from the neural crest (69). However, the survival of sensory neurons is not totally dependent on BDNF: When their axons begin to move toward peripheral targets, in fact, the neurons survive independently of BDNF. Once the appropriate synaptic connections have been established, they become sensitive to BDNF again and this response is necessary for their survival (70). Immunohistochemical analyses have shown that the expression of TrkB and its ligands increases during corticogenesis and is essential for the development and survival of cortical neurons; the absence of TrkB determines the apoptosis of numerous cellular elements in development at the level of layer II, III, V, and VI of the cerebral cortices (71).

## Multiple Sclerosis and BDNF: a Biomarker of the Disease Phases

There is a complex relationship between BDNF, and MS presumably associated with the different disease phases. BDNF levels have been shown to be globally reduced in people with MS (72). This could be correlated for instance, to a decreased neuroprotection (73). BDNF levels have been observed to increase in relapsing–remitting MS (RRMS) patients after a relapse compared to the stable phase of the disease (74). This suggests a possible role of the neurotrophic factor in remyelination from an acute inflammatory lesion as confirmed by numerous studies. Indeed, it has been found that BDNF levels inversely correlate with the number of lesions evidenced by magnetic resonance imaging (75). In addition, the tendency to demyelination is less sustained in EAE models when BDNF levels are increased (76). These data are reinforced by the fact that in RRMS patients, the levels of expression of BDNF and its receptor also increase during the relapse phases of the disease, and this event seems to constitute a biochemical pathway useful for achieving the subsequent phase of remission (77). Furthermore, TNR (Tenascin R), an activator of BDNF and modulator of microglia with a role in neuroprotection (78) and ARHGEF10${}_{,}^{-}$ involved in the processes of remyelination and neurogenesis (79), is also over-expressed during a relapse. The levels of such molecules decrease then once clinical remission is reached (77).

**Figure 2:**
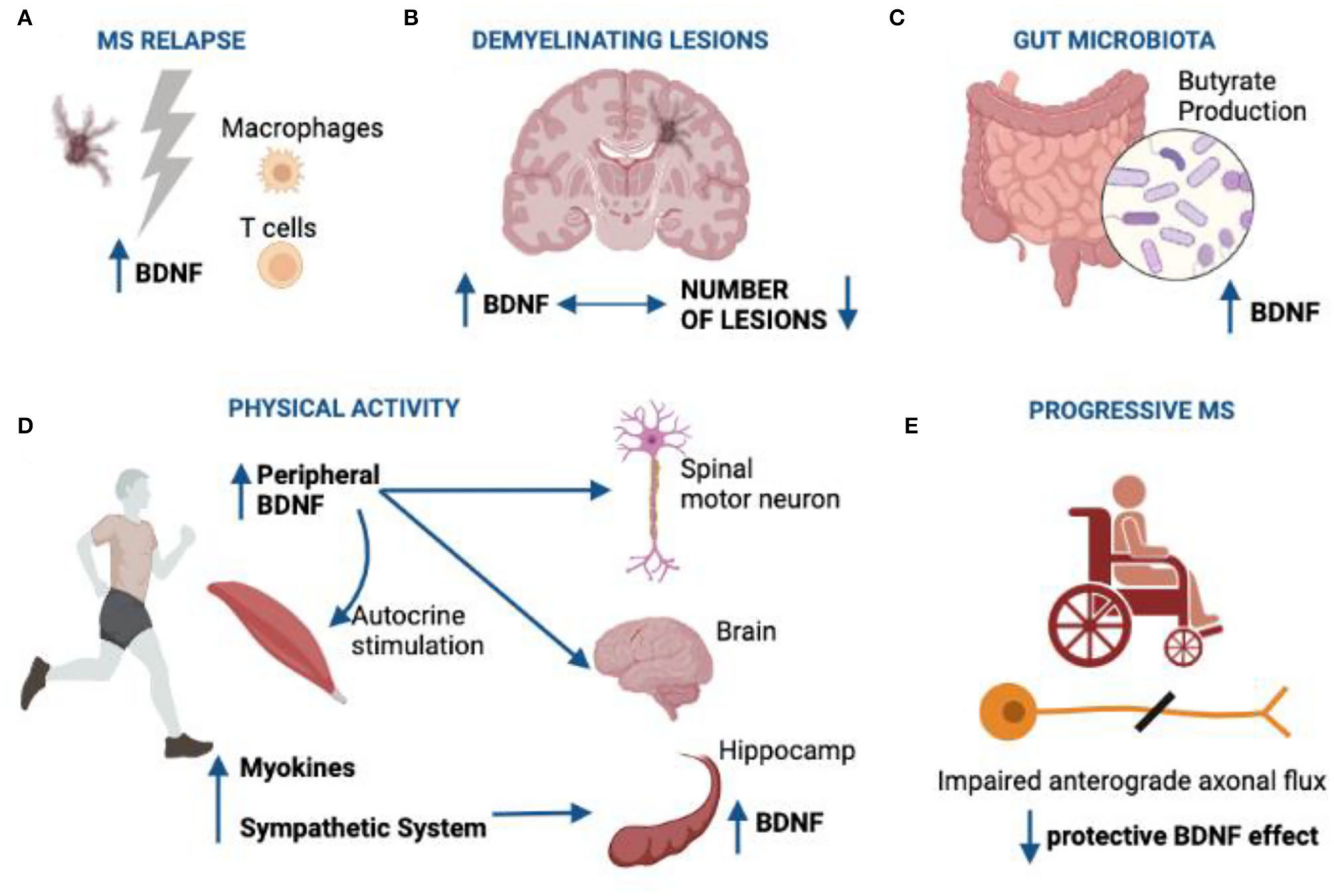
Role of BDNF in multiple sclerosis. **(A)** BDNF is reduced in patients with MS although during the relapses, its production is enhanced by neurons, macrophages, and T cells; **(B)** BDNF levels are inversely correlated with the number of lesions evidenced by magnetic resonance imaging; **(C)** gut microorganisms producing butyrate are able to induce BNDF expression; **(D)** muscles are able to produce BDNF and myokines as well as they may stimulate sympathetic system. Peripheral BDNF may directly cross the BBB, thus explaining its effect on CNS. Also, it may serve as retrograde signal for the motor neurons of the spinal cord. Myokines such as irisin and sympathetic system may both stimulate the BDNF production in hippocamp; **(E)** in progressive multiple sclerosis, the impairment in anterograde axonal flux may lead to disrupted BDNF afflux in demyelinating lesions, thus reducing the protective role of BDNF. BDNF, brain-derived neuron factor; MS, multiple sclerosis.

Immunohistochemistry analyses showed how macrophages and T lymphocytes, positive for BDNF, have been found in the active lesions of the brain in MS patients and their number correlates with the entity of demyelination. Chronic and inactive lesions are differently infiltrated by a smaller number of BDNF-secreting cells (80). The potential significance of this presence is unclear, as the BDNF produced by the SI cells seems unable to exert neuroprotection in a model of MOG_35−55_ EAE (81). However, outside the areas of injury, in MS patients and in healthy controls, neurons are the main source of BDNF. Since BDNF produced by neurons can be transported in an anterograde direction (82), its release at lesion sites in MS may provide further aid to the supportive action already performed by the BDNF released locally by immune cells. Furthermore, the anterograde transport of BDNF increases after damage to the axon (83). Conversely, in the progressive stages of disease, characterized by a deficiency in antegrade axonal transport due to the reduced expression of proteins belonging to the kinesin family (84), the efficiency of protective functions of BDNF may be lost or reduced. Figure 2 illustrates the effects of BDNF in MS.

### BDNF Polymorphisms and MS

One polymorphism in *BDNF* gene, the *Val66Met* polymorphism leading to the substitution of a methionine in place of a valine at position 66, has been observed to be associated with an alteration in activity-dependent secretion of BDNF, while the constitutive secretion of neurotrophins does not change. Subjects with the *Val66Met* polymorphism have defects in memory because of alterations in hippocampal functions (85). In Italy, in a population of 223 people examined, the *Met/Met* homozygosis is present in 4.3% of cases, while the heterozygous *Met/Val* form is present in 32.6% of cases (86). Met carriers are more numerous in Asian populations than in Caucasians (87). Studies have shown conflicting results about associations of BDNF levels in controls and subjects with this *BDNF* polymorphism. Indeed, higher (88), lower (89), and similar (90) levels of BDNF have been found in Met carriers compared to controls. In subjects with MS, in a longitudinal study conducted on a population of Southern Italy, patients with *Val66Met* polymorphism showed higher peripheral BDNF levels than healthy controls (91). Differently from what is evidenced in other neurological diseases, the *Vat/Met* polymorphism of BDNF is associated with better cognitive performance than the *Val/Val* form on neuropsychological tests (92). Furthermore, the *Val/Met* polymorphism is associated with a conservation of gray matter in MS (93) and increased hippocampus–posterior cingulate cortex connectivity (94). However, further analysis is needed. In fact, these data appear to be clearly in contrast with other observations, such as the fact that the Met allele is related to a loss of gray matter (95). In a study on Polish population, BDNF *196G/G* genotype resulted to be associated with an increased incidence of the disease, although this finding was only found in the female population under study. The onset of the disease appears to be even earlier in six subjects with the *196G/G* genotype than in subjects with the *196G/A* genotype. On the contrary, in the same population, the risk of the disease related to the *270C/T* genotype does not seem to consider the gender (96).

### Experimental Model of MS and BDNF Role

BDNF has been shown to prolong neuron survival after axotomy (97) and induce proliferation of oligodendrocytes (98). For example, BDNF enhances oligodendrocytes proliferation and development through the activation of TrkB and the MAPK pathway without involving the p75 receptor (99) and the deficiency of this neurotrophic factor reduces the proliferation of oligodendrocytes in the basal forebrain (48). The myelin proteins, such as myelin basic protein, myelin-associated glycoprotein, and proteolipid protein, are also compromised by the reduction in BDNF (100). Furthermore, the use of agonists that mimic the effects of BDNF on the TrkB receptor can induce the repair of damaged myelin (101). The effects of BDNF have been investigated in numerous animal models of MS. In cuprizone-induced demyelination, TDP6, a mimetic of BDNF, induces oligodendrocyte differentiation and myelin repair via the expression of TrkB (102). Similar effects are obtained during the process of remyelination of the corpus callosum by the TrkB Agonist LM22A-4, which increases the density of the oligodendrocytes, stimulating their myelin. Both effects are achieved by stimulating the TrkB receptor (103). Since the BDNF transport system through the BBB is saturable, methods have been recently tested to increase the delivery of BDNF into the brain thanks to the simultaneous administration of substances capable of acting on the permeability of the BBB. For example, in relapsing–remitting experimental autoimmune encephalomyelitis (RR-EAE), eight intravenous injections of BDNF (5.7 nmol/kg)—every 4 days beginning on day 21 after EAE induction, using also the ADTC5 peptide, a modulator of BBB—can induce the remyelination of the corpus callosum and suppression of relapse more in the group treated with BDNF and ADTC5 than in the group treated with only BDNF or only ADTC5 (104).

Astrocyte-derived BDNF enhances myelin repair after a demyelinating lesion (44). In cuprizone-treated mice, the injection of CHPG, an agonist of the astrocytic metabotropic glutamate receptor 5, stimulates the myelination supported by BDNF (105). The BDNF secreted by the astrocytes after the white matter lesion can also support the differentiation of oligodendrocyte precursors (106).

## Pro-BDNF and Mature BDNF in Human Blood as MS Biomarkers: Some Considerations and Limitations

Thanks to the growing literature evidence, in part abovementioned, the BDNF can represent a promising biomarker for MS disease, even if some considerations and limitations must be underlined. First, it is imperative to consider that BDNF exists in two forms, as precursor, the pro-BDNF, and mature protein, the mature BNF, having different roles and effects on neuronal survival, cognition, and myelination. Another limitation is due by the difficulty of quantifying the individual circulating levels of precursor and mature BDNF by using commercial ELISA kits. These aspects are briefly described and discussed in the following paragraphs.

### The Different Effects and Roles of Pro-BDNF and Mature BNF on Neuronal Survival, Cognition, and Myelination

Diverse roles and effects appear to mediate the pro-BDNF and mature BDNF. Precisely, pro-BDNF levels increase in aged mice brains, while BDNF transcripts decrease during aging (107). Increased levels of pro-BDNF at the hippocampal level are associated with memory loss (108) and intra-hippocampal administration of anti-proBDNF antibodies reduces cognitive dysfunction (109). Moreover, synaptic plasticity dysfunctions are mediated by p75^NTR^, a receptor typically activated by pro-BDNF (110). Differently, BDNF, through the TrkB receptor, helps to strengthen synapses and has a positive role in memory and cognition (111). Yet, neuronal survival is negatively affected by pro-BDNF, which can induce cell death in neurons through the co-stimulation of p75^NTR^ and sortilin (112). Interestingly, truncated TrkB isoforms may also contribute to hindering the BDNF signaling (113).

The role mediated by the BDNF/pro-BDNF balance on oligodendrocytes and astrocytes is also different. Oligodendrocytes express both TrkB and p75^NTR^, suggesting that these cells are sensitive to mature BDNF and pro-BDNF (48). Pro-BDNF appears to have inhibitory effects on oligodendrocytes. Indeed, pro-BDNF reduces cell proliferation and migration in a line of OLN-93 oligodendrocytes through p75^NTR^ signaling (114). Furthermore, the expression of p75^NTR^ does not seem to be required to induce myelination. In fact, only the BDNF promotes myelination and proliferation in cultures of oligodendrocytes (115) and BDNF-deficient mice have reduced levels of various myelin proteins, such as MAG, MBP, and PLP, and a deficit in the production of the oligodendrocyte lineage (47). Furthermore, loss of BDNF appears to specifically affect oligodendrocytes but not astrocytes or microglia (47). However, myelination appears to be an effect mediated directly by the TrkB receptor, while the proliferation of OPC is independently influenced by TrkB, because it can be stimulated by the expression of TrkC (116).

Astrocytes are also targets for both BDNF and pro-BDNF. Pro-BDNF contained in extracellular vesicles derived from astrocytes has negative effects on cell survival (117), and p75^NTR^ stimulation is associated with oligodendrocytes loss after a spinal cord damage (118). Neuronal death induced by isoflurane is reduced by the buffering of pro-BDNF levels induced by the intervention of astrocytes (119). Following a damage to myelin, the BDNF capable of supporting myelination is not only that produced by oligodendrocytes but also that produced by astrocytes (44). In fact, the BDNF-secreting astrocytes can induce the transition from the immature form of the oligodendrocytes to the myelin-producing phenotype. The analogous differentiation in microglia, on the contrary, seems to be supported by IGF-1 and not by BDNF (120). The neurons themselves, after damage to myelin, release an action potential that stimulates the release of BDNF along the exposed axon. This stimulates the OPCs near the axon to differentiate into mature oligodendrocytes, to proliferate, and to promote the activity-dependent myelination (43).

### Levels of the Two Form BDNF in Human Blood and Cerebrospinal (CSF) Samples: The Current Evidence

The accurate measurement of blood BDNF levels could serve as a potential biomarker of MS, given its presence in circulation, and even if highly concentrated in brain tissue. Of further relevance would be the quantification of the individual circulating levels of precursor and mature BDNF, given the different effects mediated. The BDNF levels in human blood can be assessed by using commercially available human BDNF ELISA kits. However, the reduced specificity of the BDNF antibodies of these kits makes difficult to discriminate the circulating pro-BDNF amount than that of mature BDNF, also using human pro-BDNF or BDNF ELISA kits. This question has been raised by Yoshida and coworkers in 2012 (121), by quantifying the serum levels of precursor and mature BDNF in healthy subjects. Precisely, they have observed an unacceptable sensitivity of pro-BDNF kit (121). Consequently, the development of highly sensitive pro-BDNF and BDNF ELISA systems, as well as of standardized methodologies and measures currently represent a priority. In addition, it is recently considered to use other fluid liquids as more appropriate biological matrices for testing the BDNF levels or combining different evaluations in the same biological liquid. For example, interesting results have been obtained by assessing the levels of BDNF and neurofilament light chains (NfL) in both serum and cerebrospinal (CSF) samples of 42 newly diagnosed MS patients (122). Precisely, the group of Dr. Foerch has evidenced that CSF BDNF and NfL levels measured at the time of the diagnosis appeared inversely associated with cognitive performance in MS cases (122). Therefore, such suggests that CSF biomarkers related to different pathophysiological processes can indicate neuropsychological impairment in the earliest stages of the disease, and consequently, a combination of different CSF measures might facilitate the developing of a better biomarker of cognition in MS (122).

## Strategies for Improving the Pathophysiology of MS

### Physical Activity and BDNF

Physical activity in MS patients has been shown to have numerous beneficial effects, being able to improve, for example, the gait stability and walking (123, 124). A single exercise session can increase BDNF and NGF levels measured in the periphery (125, 126), and physical exercise can have positive effects on symptoms progression in MS patients (126). In fact, the muscles in activity produce and release BDNF into the circulation apparently involved in the first place in the autocrine and paracrine stimulation of the muscles themselves (127, 128). An interesting hypothesis is that BDNF coming from the muscles also influences the brain; in fact, it has been shown that the BDNF can cross the blood–brain barrier (BBB) in both directions, thanks to a high-capacity and saturable transport system (129). Therefore, peripheral blood BDNF levels could mirror the amount of neurotrophic factor present in the brain (130), and peripheral BDNF measurements performed following completion of motor exercise programs can provide comparable values to the concentrations present in the nervous system. The molecular mechanisms by which physical exercise activities stimulate the production of BDNF are multiple. For example, in mice a myokine, cathepsin B, produced following muscle training, is capable of positively influencing neurogenesis through the production of BDNF (131). Myokines are molecules able to cross the BBB and directly stimulate the synthesis of BDNF at the hippocampal level (132). Accordingly, the group of Dr. Y Wang has recently evidenced that the muscle acts as a secretory organ, by producing myokines, having the critical role to communicate with other organs, such as the brain, and evocating the increase in the brain levels of BDNF (133). For example, the irisin is another myokine produced following physical exercise, which can increase the production of BDNF at the hippocampal level through the mediation of PGC-1α (134).

BDNF is therefore configured as a factor capable of mediating the positive effects of physical activity on cognitive changes. Study investigation the effects of exercise seem to confirm this hypothesis. Aerobic exercise activity conducted for more than 3 months by subjects with MS and volunteers showed an increase in hippocampal volume (135), and physical activity has been shown as well to increase BDNF concentrations in subjects with multiple sclerosis (136). The effects of physical exercise on the levels of BDNF and other neurotrophic factors are independent of disability status (137). Muscle tissue, itself, participates indeed in the production of BDNF. This, in fact, can behave like a protein whose synthesis is inducible by contraction by the active muscle and is aimed at inducing, among other things, lipid oxidation in the muscle (137). It has also been shown that IS cells produce BDNF through stimulation effects from physical activity (138). Finally, muscle exercise is accompanied by the activation of the sympathetic nervous system which, through adrenergic endings, stimulates the expression of hippocampal BDNF and reduces the production of inflammatory cytokines. All this constitutes an important suggestion that the functions of stimulating growth, proliferation, and neuronal survival regularly performed by BDNF can be, in some way, elicited by physical activity. Not surprisingly, when the interaction between the TrkB receptor and BDNF is blocked, the beneficial effects of rehabilitating physical exercise following damage to the spinal cord are also lost (139).

### Probiotics for Recovering BDNF Expression and Ameliorating the Clinical Conditions Related to MS

Alterations in the expression of BDNF are associated with the pathophysiology of MS, as above largely described. Consequently, the recovery of such BDNF expression might represent an advantageous therapeutic MS strategy. In this context, it appears relevant the recent indication of a possible beneficial effect of butyrate production by gut microorganisms in inducing BNDF expression and activating its secretion (140–143). In addition, the group of Romo-Araiza has also shown in middle-aged rats how probiotic and prebiotic supplementation significantly better than other groups improve the spatial memory, by increasing the BDNF levels. Such strategy, whether confirmed by clinical studies, might be used as potential therapy to be associated with current disease-modifying strategies in MS. Currently, only a small number of studies have tested the probiotic supplementation in individuals with MS (143–145). In the two small double-blinded randomized controlled trials, cases with MS daily received for 12 weeks a mix of *Lactobacillus* and *Bifidobacterium* supplementation and showed significant improvements in their disability score, depression, anxiety, and inflammatory markers (143, 144). In another study, the group of Tankou and coworkers used a probiotic mix with *Lactobacillus, Bifidobacterium*, and *Streptococcus* administered daily for 2 months to MS patients (145). Results of these studies reported a diminished CD80 expression on peripheral monocytes, which was anyway not sustained after probiotic discontinuation, thus suggesting the necessity of continuous supplementation (145) We conclude, hence, that there is no at present time, clear evidence of the biological effects of such supplementation on BDNF expression, secretion, and levels.

Another promising evidence arrives from the data, obtained by Chinese researchers (146), about the treatment with antidepressant (R)-ketamine in cuprizone (CPZ)-treated mice and remyelination after CPZ withdrawal. Precisely, they have recently demonstrated that the repeated treatment with (R)-ketamine (10 mg/kg/day, twice weekly, for 6 weeks) significantly ameliorated demyelination and activated microglia in the brain compared with saline-treated mice (146). In addition, it has been shown that the pretreatment with a TrkB antagonist, the ANA-12, significantly inhibited the beneficial effects of (R)-ketamine on the demyelination and activated microglia in the brain of CPZ-treated mice. The 16S rRNA evaluation has also revealed that (R)-ketamine significantly ameliorated the altered composition of gut microbiota and reduced the levels of lactic acid in CPZ-treated mice. Accordingly, significant correlations between the demyelination (or microglial activation) in the brain and the relative quantity of several microbiomes were detected, suggesting a close relationship between gut microbiota and brain. These relevant results have led the Dr. X Wang group to suggest that (R)-ketamine could likely be a new therapeutic drug for MS (146).

## Conclusion

This overview on BDNF evidences its role in multiple physiological processes and diseases, particularly in MS, as above reported. Accordingly, compelling evidence demonstrates that impairment in the synthesis and levels of BDNF and its signaling are related to diverse pathologies, such as MS. Depending on the pathological conditions, such alterations can be responsible for damaging modifications in synaptic transmission, plasticity, neuronal survival, and cognitive performance maintenance, which also reflects the importance of this neurotrophic system.

The relevant importance of BDNF-related metabolic pathways, in the onset of MS, has led to suggest its role as a potential biomarker, as well as in some therapeutic strategies including physical exercise, the use of probiotics, or more recently the administration of BDNF itself. This last promising perspective has some limitations characterizing it, such as the inability to cross the BBB, the reduced half-life of the molecule, and potential adverse side effects. In addition, the possibility to delivering BDNF in a specific brain region represents another crucial limitation. Consequently, the two mentioned ways, that is physical exercise and the use of probiotics, or likely other indirect treatments able at promoting or re-establishing BDNF signaling and physiological levels, might be used.

However, it is possible to affirm that no effective MS treatments do exist until now, and a major knowledge of the mechanisms and pathways involved in BDNF dysfunction is essential to develop suitable strategies for the MS pathological scenario, as well as to suggest BDNF dysfunction as potential MS biomarker useful in early diagnosis and prognosis.

## Author Contributions

CB contributed to conceptualization and supervision. GSc and CB wrote the manuscript. PA, GSc, and SI performed the search and validation of literature data. GSc and SI contributed to drawing figures. CB, PR, and GSc contributed to the validation, revision, and editing. All authors contributed to the article and approved the submitted version.

## Funding

This work was funded by Italian University Minister FFABR grant 2017 to CB and University of Palermo FFR 2019/20 grants to CB.

## Conflict of Interest

The authors declare that the research was conducted in the absence of any commercial or financial relationships that could be construed as a potential conflict of interest.

## Publisher's Note

All claims expressed in this article are solely those of the authors and do not necessarily represent those of their affiliated organizations, or those of the publisher, the editors and the reviewers. Any product that may be evaluated in this article, or claim that may be made by its manufacturer, is not guaranteed or endorsed by the publisher.
